# A systematic review of experimental evidence on microbial pathogen transmission by *Stomoxys* spp.

**DOI:** 10.1051/parasite/2026014

**Published:** 2026-03-19

**Authors:** Metlholo Andries Phukuntsi, Maropeng Charles Monyama, Moeti Oriel Taioe, Ana Mbokeleng Tsotetsi-Khambule

**Affiliations:** 1 Department of Life and Consumer Sciences, College of Agriculture and Environmental Sciences, University of South Africa Johannesburg GP South Africa; 2 Department of Epidemiology, Parasites and Vectors, Agricultural Research Council – Onderstepoort Veterinary Research, Onderstepoort Pretoria GP South Africa

**Keywords:** Pathogens, *Stomoxys*, Host, One Health, Human-livestock-wildlife, Transmission

## Abstract

Vector-borne microbial pathogens previously isolated from *Stomoxys* spp. are currently considered to be emerging or re-emerging threats to public health and the veterinary sector. Transmission of pathogens by flies in the *Stomoxys* genus is largely mechanical, indicating that they can transmit a wide range of pathogens to a variety of hosts. This study evaluated the diversity of pathogens demonstrably transmitted by a variety of *Stomoxys* flies, concerning species diversity, host diversity, and geographic distribution. Preferred Reporting Items for Systematic reviews and Meta-Analyses (PRISMA) guidelines were applied to screen studies based on pathogen type, host species, and experimental transmission outcomes. Journal articles published from 1973 to 2025 were sourced from six electronic databases. After evaluation, 30 studies were eligible for this review. Of these studies, 20% (6/30) reported negative outcomes. Three pathogens (Middle East respiratory syndrome coronavirus, *Neorickettsia risticii*, and *Escherichia coli*) were not transmitted by the flies in the experiments. *Stomoxys* spp. transmitted pathogens to a wide range of hosts (9 mammals) and substrates (blood and tissue culture), but the recorded experiments in camels failed. Three out of ten *Stomoxys* spp. reported in the studies (*S. transvittatus, S. inornatus*, and *S. omega*) failed to transmit pathogens in all attempts. The majority of experimental studies were on *S. calcitrans,* with very limited studies on other *Stomoxys* species, highlighting the dearth of information on other species occurring in Africa and Asia. Our study has consolidated the evidence regarding the experimental pathogen transmission by *Stomoxys* spp., highlighting and demonstrating their epidemiological significance and the need for surveillance and control/prevention strategies.

## Abstract (fr)

Les agents pathogènes microbiens transmis par des vecteurs et précédemment isolés chez les *Stomoxys* spp. sont actuellement considérés comme des menaces émergentes ou réémergentes pour la santé publique et le secteur vétérinaire. La transmission de ces agents pathogènes par les stomoxes est principalement mécanique, ce qui indique leur capacité à transmettre un large éventail d’agents pathogènes à divers hôtes. Cette étude évalue la diversité des agents pathogènes transmis par différentes espèces de stomoxes, en considérant la diversité des espèces, la diversité des hôtes et la distribution géographique. Les recommandations PRISMA ont été appliquées pour sélectionner les études en fonction du type d’agent pathogène, de l’espèce hôte et des résultats expérimentaux de transmission. Les articles publiés entre 1973 et 2025 ont été extraits de six bases de données électroniques. Après évaluation, 30 études ont été retenues pour cette revue. Parmi celles-ci, 20 % (6/30) ont rapporté des résultats négatifs. Trois agents pathogènes (le coronavirus du syndrome respiratoire du Moyen-Orient, *Neorickettsia risticii* et *Escherichia coli*) n’ont pas été transmis par les mouches lors des expériences. Les espèces de *Stomoxys* ont transmis des agents pathogènes à un large éventail d’hôtes (9 mammifères) et de substrats (cultures de sang et de tissus), mais les expériences réalisées sur des chameaux ont échoué. Trois des dix espèces de *Stomoxys* recensées dans les études (*S. transvittatus*, *S. inornatus* et *S. omega*) n’ont pas réussi à transmettre d’agents pathogènes lors des différentes tentatives. La majorité des études expérimentales ont porté sur *S. calcitrans*, les études sur les autres espèces de *Stomoxys* étant très limitées, ce qui souligne le manque d’informations sur les autres espèces présentes en Afrique et en Asie. Notre étude a consolidé les données probantes concernant la transmission expérimentale d’agents pathogènes par les espèces de *Stomoxys*, mettant en évidence leur importance épidémiologique et la nécessité de mettre en place des stratégies de surveillance, de contrôle et de prévention.

## Introduction

The genus *Stomoxys* comprises 18 species and is classified under the family Muscidae and order Diptera in Arthropoda [16]. Both sexes of the *Stomoxys* spp. are hematophagous and use small prestomal teeth to pierce the skin and a proboscis to suck blood [65]. *Stomoxys* spp. are major pests and vectors of pathogens and they play a major role in the emergence and re-emergence of zoonotic diseases across the world [13]. In the last decade, diseases caused by fly-borne pathogens, previously detected in *Stomoxys* spp., such as African swine fever and besnoitiosis, have been increasingly documented in areas where they had not been reported before or in recent years, and are considered emerging or re-emerging threats [2, 52, 21].

A variety of microbial pathogens that include protists, bacteria, and viruses have been isolated from *Stomoxys* spp. These include, but are not limited to, agriculturally important protozoan pathogens such as *Trypanosoma* spp., *Babesia* spp., and *Besnoitia* spp. (causative agents of nagana, babesiosis, and besnoitiosis, respectively), bacterial pathogens such as *Anaplasma marginale* (causative agent of anaplasmosis) and viral pathogens such as lumpy skin disease virus (LSDV) and African swine fever virus (ASFV) [1, 41]. Host defence response due to painful bites interrupts feeding, leading to the fly finding an alternate host and thus increasing the likelihood of spreading pathogens within herds in a relatively short time. Dispersal in the species is suspected to be reliant on available hosts. Therefore, they may travel great distances to find hosts [65], thus increasing the likelihood of expansions of local outbreaks of diseases.

*Stomoxys* spp. are largely associated with the agricultural sector, where they occur in high abundance in areas with livestock activity, including feedlots, slaughterhouses, and markets [60]. They have also been found at other sites with a wide range of land-use, including national parks and other conservation areas, pasture areas, and villages [16]. Except for *Stomoxys calcitrans* [39], the stable fly, a cosmopolitan species found across all major biogeographic realms, except for Antarctica [16], species within *Stomoxys* are tropical and variably distributed across Africa and Asia [16, 15]. Fourteen of the species are found in Afrotropical bioregions, three species in Reunion Island and six species in the Indomalaya bioregions (Supplementary Table S1).

The last comprehensive review of the flies in the *Stomoxys* genus, as well as their impact and transmission of pathogens, was undertaken by Zumpt [74]. Since then, the last review of pathogen transmission by *Stomoxys* was undertaken and published by Baldacchino *et al.* [1], who documented mechanical and biological transmission of viral, bacterial, protozoan, and helminth pathogens established through natural, experimental, and isolation studies. Since the review of Baldacchino *et al.* [1], there have been major emerging and re-emerging diseases across the world that have been identified from *Stomoxys* spp., such as African swine fever (ASF), besnoitiosis, and lumpy skin disease [2, 21, 40, 51]. We therefore performed this review to determine the diversity of the microbial pathogens that have been experimentally transmitted by *Stomoxys* flies, looking at the diversity of the experimental hosts and the geographic distribution of the *Stomoxys* spp. that experimentally transmit the pathogens.

## Materials and methods

### Study design

The systematic review was conducted using the recommended method as outlined in the Preferred Reporting Items for Systematic Reviews and Meta-analyses (PRISMA) guidelines [53]. The following procedures were followed during the review: (i) articles that might be relevant were classified using database searches, (ii) assessments of the articles’ applicability to the review, (iii) assessments of the quality of relevant articles, and (iv) data extraction, screening, and analysis.

### Review questions

This systematic review was conducted to synthesise the outcomes of studies on experimental microbial pathogen transmission by *Stomoxys* spp. The objectives of the review were to determine the diversity of the pathogens that have been demonstrably transmitted by *Stomoxys* flies through experimental designs, the diversity of the hosts, and the geographic distribution of the *Stomoxys* spp. that experimentally transmit the pathogens. The research question of this review is “What was the diversity of pathogens demonstrably being transmitted by flies in the *Stomoxys* genus, and What was the host diversity of the flies?”

### Systematic search strategy and selection criteria for journal articles

Following the endorsed methodology by the PRISMA guidelines [53], keywords were used to retrieve studies from electronic databases, namely, PubMed (https://pubmed.ncbi.nlm.nih.gov), Science Direct (https://www.sciencedirect.com), Google Scholar (https://scholar.google.com), Semantic Scholar (https://www.semanticscholar.org), OpenAlex (https://openalex.org), and Scopus (https://www.scopus.com). The search was conducted independently by two researchers (MAP and CMM), both manually and using the program “Publish or Perish” [22]. The search was conducted between March (MAP) and April (CMM), 2025. The systematic search was accomplished following the combination of free text and wordlist terms in diverse distinctions: “*Stomoxys*”, “*Stomoxys* spp.”, “transmission”, “pathogen”, “bacteria”, “bacterial”, “protozoa”, “protozoan”, “virus”, and “viral”, using the Boolean operators “AND” and “OR”. Thereafter, titles and abstracts were assessed, and potential articles were reviewed and downloaded for analysis. The reference lists from the potentially searched published articles were also screened for articles relating to the review. The following criteria were used to narrow the final list of articles: (a) duplicate articles were checked and removed from consideration, (b) articles that did not meet the inclusion criteria were also removed, (c) articles that did not fit the aim and scope were not taken into consideration, and (d) articles published before 1973 were not included in this systematic review.

### Eligibility criteria

Records from the electronic databases were initially identified using the title and abstract. To determine eligible journal articles for the review, all retrieved articles that were relevant based on their titles were further screened based on their abstracts. When the emphasis was not on flies in the *Stomoxys* genus, the fly hosts and their associated pathogens, the records were typically excluded. Theses, dissertations, and records with unclear methodology were also removed.

### Inclusion criteria

The inclusion criteria were peer-reviewed studies published in English from 1973 to 2025, whose objectives included explicit experimental demonstration of transmission of bacterial, protozoan, and/or viral pathogens by *Stomoxys* spp., where transmission is determined by the presence of the pathogen in the vector and host within a prescribed period and location. Inconclusive studies where transmission can neither be confirmed nor rejected and studies with negative outcomes (where the hypothesis of transmission is rejected) were also included. Only studies published in peer-reviewed journals were considered for final selection.

### Exclusion criteria

As evidence of transmission of pathogens was the explicit objective, the studies that focused on isolation of pathogens from the vector or host, by any means (culturing, cloning, molecular, *etc.*), without linking the pathogen to both the host and the vector, were excluded.

### Identifying bias risk

Thirty articles were included and analysed for quality to avoid the risk of bias. Included articles were analysed according to the JBI critical appraisal checklist (JBI Evidence Synthesis Manual) and guidelines in the gradebook and Cochrane handbook for systematic reviews of interventions [26]. The risk assessment was conducted by evaluating the following variables: (i) study location, (ii) year of publication, (iii) pathogen type, (iv) pathogen name/species, (v) sample size, (vi) detection method, (vii) specific gene detected, (viii) experimental design, (ix) transmission outcome, and (x) other present vectors.

The quality of relevant studies was evaluated by a system of scoring through a modified checklist that was based on the following questions: (a) Was the title and abstract of the study relevant? (b) Were the methods and data analysis of the study clear and sufficiently covered? (c) Does the study give a detailed description of the statistical approach and analysis? (d) Was the sample size acceptable? (e) Was the interpretation of the results and discussion considering the aim/objectives of the study? (f) Were the vector and host samples randomly selected from the population? (g) Were the vector, host, and pathogen(s) clearly identified and allocated? (h) Was the study/experimental design ethically acceptable? and (i) Were the transmission outcomes clearly stated? The assessment carried out on every paper was based on these nine questions and scored at 9/9 × 100% (Supplementary Table S2). A score above 70% was signified as a high-quality journal article, between 50% and 69% an average-quality journal article, and below 49% a low-quality journal article. Thus, no paper was omitted from this review manuscript based on the scores, since any score obtained reflects the quality of the study. Eligibility, inclusion, quality evaluation, and data extraction were all handled by two reviewers throughout the systematic review process.

### Data collection and analysis

For the analysis, data were extracted from all the eligible studies. Data were extracted manually by MAP and CMM. The following variables were extracted and documented in an Excel spreadsheet: author and publication year, year of study, fly species, host species, pathogen species, pathogen type, study type, experimental design, vector sample size, host sample size, detection method, geographic location (at three levels), and outcome of the study. The dataset was cleaned and formatted in Excel and analysed using Excel and the R programme.

## Results

### Literature search and outcomes

**Figure 1:**
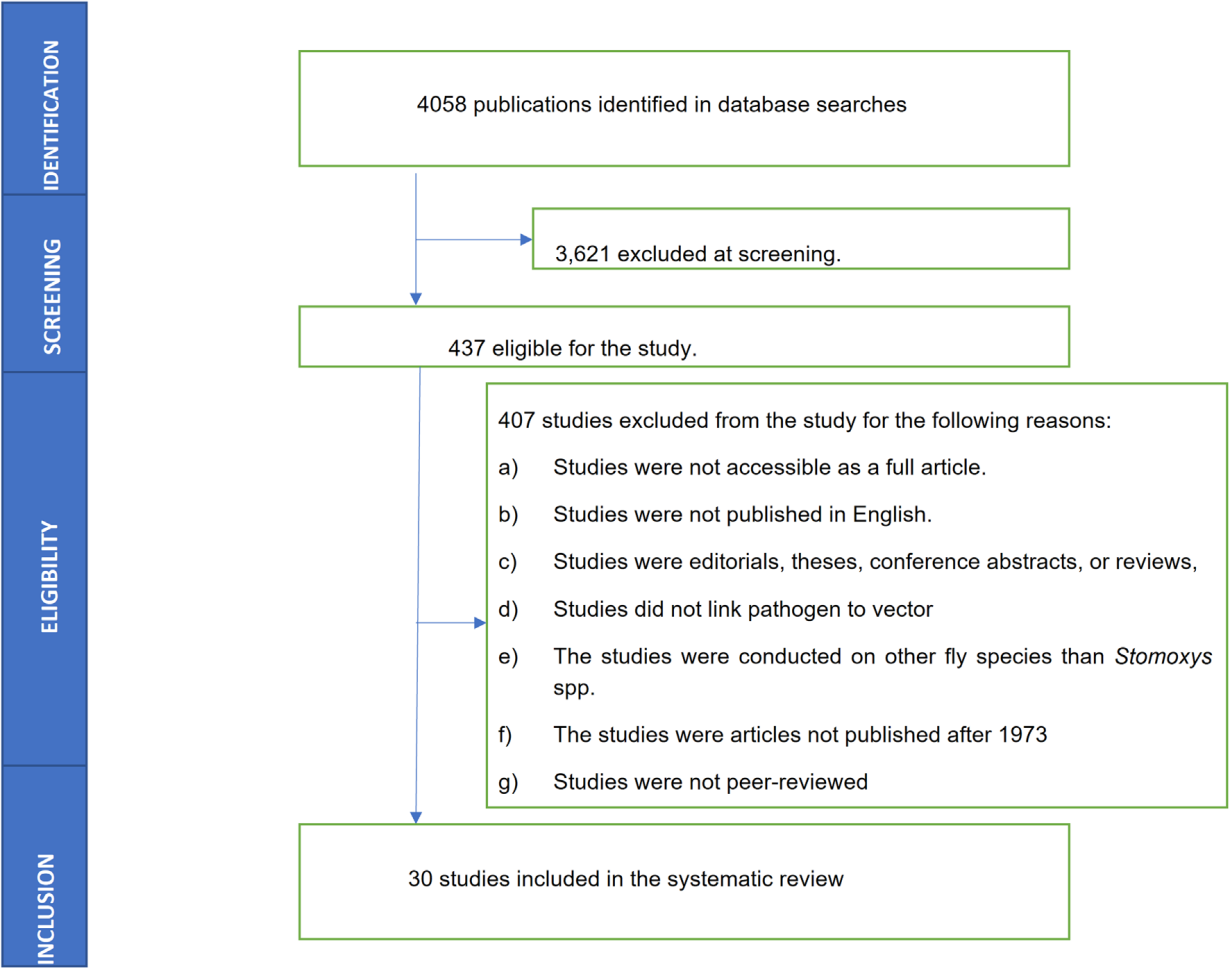
PRISMA flow diagram showing the process of finding and choosing published articles for the *Stomoxys* spp. diversity involved in the transmission of pathogens.

The literature search yielded a total of 4,058 articles from the six databases that were queried. After elimination based on the title, eligibility, exclusion and inclusion criteria, 30 articles were selected for analysis (Fig. 1).

### Quality assessment of studies

Based on a quality score between 67% and 100%, all studies included in this systematic review were deemed to have a low risk of bias (Supplementary Table S2).

### Characteristics of studies in the systematic review

The geographic distribution of studies conducted in this review is summarised in Table 1. The majority of studies were conducted in the European and North American regions. The other regions (Asia, Africa, and South America) represented fewer than half of the studies conducted. Seven of the studies did not report on the region of interest. Diversity of the species studied is also summarised in Table 1. The total number of studied species was 12. *Stomoxys calcitrans* was studied in all regions. Two species, *S. sitiens* and *S. indicus*, were studied in Asia. The rest of the species were studied only in Africa.

**Table 1:** Characteristics and summary of eligible studies on experimental transmission of microbial pathogens by *Stomoxys* spp.

Geographic region	Country	Species name	Pathogen name	Host	Reference
Africa	Cameroon, Kenya, South Africa	*Stomoxys calcitrans*, *S. niger niger*, *S. omega*, *Stomoxys n. bilineatus*, *S. pallidus*, *S. transvittatus*, *S. taeniatus*, *S. inornatus*, *and S. varipes*	Foot-and-mouth disease virus, *Trypanosoma brucei*, *T. congolense*, *T. evansi*, *T. vivax*, *Anaplasma marginale*	Cow, mouse, camel	[37, 43, 49, 56, 67]
Asia	Middle East, Kazakhstan	*Stomoxys calcitrans, S. sitiens, S. indica*	Middle East respiratory syndrome, coronavirus, Lumpy skin disease virus	Camel, cow	[25, 30]
Europe	Romania, Belgium, England, France, Denmark	*Stomoxys calcitrans*	African Swine Fever Virus, Lumpy skin disease virus, Capripox virus, *Besnoitia besnoiti*	Pig, cow, sheep, sterile blood, rabbit	[2, 21, 34, 38, 51, 63, 64, 66]
North America	USA, Canada	*Stomoxys calcitrans*	*Ehrlichia risticii*, Equine infectious anaemia virus, *Staphylococcus aureus*, *Anaplasma marginale*, Rift Valley Fever Virus	Pig, mouse, horse, sterile blood, cow	[4, 23, 47, 59, 62, 70]
South America	Brazil	*Stomoxys calcitrans*	*Escherichia coli*, *Trypanosoma vivax*	Cow	[7, 24]
Unknown		*Stomoxys calcitrans*	Bovine leucosis virus, Equine infectious anaemia virus, Bovine herpes mammillitis, Capripox virus, African Swine fever virus, *Eperythrozoon suis*, Porcine Reproductive and Respiratory Syndrome Virus*, Bacillus anthracis,* Bovine leukaemia virus	Sheep, cow, horse, tissue culture, goat, pig, mouse, guinea pig	[5, 18,19, 42, 57, 69, 73]

Only 10% (3/30) of studies incorporated *in vitro* hosts, namely, cell culture and sterile blood (Supplementary Table S4). The host range of *Stomoxys* spp. included wildlife species (rabbit), domesticated wildlife species (mice and guinea pigs), and domesticated animals (camel, cow, goat, horse, pig, and sheep) (Table 1). The diversity of pathogens in experimental transmission by *Stomoxys* spp. is summarised in Table 1. The pathogens studied in Africa were the *Trypanosoma* spp. (East Africa), *A. marginale* (southern Africa) and Foot and mouth disease virus (FMDV) in Central Africa. In Western Europe, the pathogens studied were ASFV, LSDV, *Capripox* virus, and *Besnoitia besnoiti*. In Eastern Europe, the Middle East, and Central Asia, the studied pathogens were ASFV, Middle East respiratory syndrome coronavirus, and LSDV, respectively. In South America, the pathogens studied were *E. coli* and *T. vivax*. In North America, pathogens studied were *S. aureus*, *A. marginale*, *N. risticii*, Porcine Reproductive and Respiratory Syndrome Virus, and Rift Valley Fever Virus.

Of the thirty reviewed studies, four studies (13%) used a field approach, one each (3%) allowed the flies to feed on cultured tissue medium or be ingested by the host, three (10%) allowed the flies to feed on sterile blood substrate, two (7%) injected fly material into the host, while 22 (73%) allowed the flies to feed on the host (Supplementary Table S3).

### Experimental microbial pathogen transmission by *Stomoxys* spp.

#### Species and host diversity in the transmission of pathogens by *Stomoxys* spp.

Of the thirty studies eligible in this review, 21 (70%) reported at least one positive outcome for demonstration of the transmission of a pathogen by *Stomoxys* spp., six (20%) reported negative outcomes (no transmission), while three (10%) were inconclusive (Supplementary Table S4). Based on the data from these studies, *S. calcitrans* can transmit a wide variety of bacterial, protozoan, and viral pathogens to a wide range of hosts in experimental settings. *Stomoxys calcitrans* successfully transmitted bacterial, protozoan, and viral pathogens in 20 of the 29 studies conducted, on pathogens occurring on all five continents. *Stomoxys calcitrans* was able to transmit pathogens to all but one livestock species studied (pigs, cows, sheep, and goats), with the exception of camels. Furthermore, *S. calcitrans* was able to transmit pathogens to domesticated wildlife species, namely, guinea pigs, mice, and rabbits. Most notably, this species successfully transmitted known re-emergent viral diseases in Europe and America (LSDV, ASFV, and RVF). In addition to this, the species also demonstrated the ability to transmit *B. besnoiti*, a known re-emergent protozoan pathogen in Europe, to a cow and a rabbit. Thus, the data in this review further highlight the risk of *S. calcitrans* in transmitting a variety of pathogens in livestock and wildlife globally, with an increased risk of spreading re-emerging or introduced pathogens.

Aside from *S. calcitrans*, seven other species can experimentally transmit pathogens. The largely Asian species, *S. indicus* and *S. sitiens*, were shown to successfully transmit LSDV to a cow. Thus, in Asia, *S. calcitrans* is not the only *Stomoxys* spp. posing a transmission risk and spread for this virus. Notably, *S. niger niger*, the most ubiquitous species in Africa, could successfully transmit multiple *Trypanosoma* species, including *T. evansi*, *T. congolense* and *T. vivax*, although experiments were unsuccessful for *T. brucei*. This demonstrates that in Africa, *S. n. niger* poses a high risk as a vector of these *Trypanosoma* pathogens. Other *Stomoxys* spp. that can transmit *Trypanosoma* spp. in an experimental setup are *S. bilineatus* (*T. brucei* and *T. vivax*), *S. pallidus* (*T. brucei*), *S. taeniatus* (*T. brucei*), and *S. varipes* (*T. brucei* and *T. evansi*). However, there appears to be variation among these species regarding transmission risk of various *Trypanosoma* species, although *T. brucei* demonstrates a higher flexibility among the species.

#### Other factors governing transmission of pathogens by *Stomoxys* spp.

While *Stomoxys* spp. demonstrate the ability to transmit a wide variety of bacterial, viral, and protozoan pathogens, not all exposures are successful. The transmission success in this review ranged from 0% to 100%, and this varied within and between pathogens, hosts and species, as well as between time of exposure after feeding. However, due to the wide variation in methodology and reporting of results, no summary statistics are reported. As such, notable observations are highlighted here. The lowest success rate in terms of positive results was 20% in bacteria and 2.4% in protozoa. *Stomoxys* spp. transmitted a variety of pathogens from immediately after feeding to a least a day after feeding. This was demonstrated in bacterial (*S. aureus*), protozoan (*B. besnoiti*), and viral (Capripox virus) pathogens. While there is not much information on the length of time after feeding at which the flies can no longer transmit pathogens, there is at least an indication that time after exposure plays a role in transmission of pathogens by the flies. The ability of the *Stomoxys* spp. to transmit pathogens was shown to diminish with time after exposure in *Mycoplasma suis* (1 h), *Bacillus anthracis* (24 h), and *B. besnoiti* (48 h) after initial successful transmission. Negative outcomes in the transmission of pathogens were reported for three pathogens, namely, Middle East respiratory syndrome coronavirus, *N. risticii*, and *E. coli*.

Interestingly, *Stomoxys* spp. were also able to transmit pathogens by other means aside from feeding on one host to another. Of note, *S. calcitrans* was able to transmit by being ingested by hosts, by hosts being infected by fly material, as well as by feeding on substrates.

## Discussion

*Stomoxys* spp. are some of the most impactful biting flies, greatly affecting not only livestock but wildlife as well. This genus is very diverse and has an extensive range of hosts. Its impact on livestock has been widely reviewed (*e.g.*, [1, 8, 16, 68, 74]. Recently, interest in the role of the *Stomoxys* flies in pathogen transmission has been renewed, considering emerging, re-emerging and range expansion of vector-borne diseases in various regions globally. This systematic review synthesised and assessed data from relevant studies on the transmission of microbial vector-borne pathogens by *Stomoxys* spp. This review highlights the diverse pathogens demonstrably transmitted by various *Stomoxys* spp. globally.

### Species diversity

This genus is taxonomically diverse; however, most studies focused on *S. calcitrans* as it is a cosmopolitan species. The current study highlighted that *S. calcitrans* is the most researched species regarding pathogen transmission. This is not surprising as it is the only species that occurs outside the Indomalaya and Afrotropical regions [16]. The African continent was the most diverse region studied in terms of the number of species, which is also the most diverse region in terms of the number of known species [16]. Africa was also the only region where another species, *S. n. niger* (3/4 studies) was more represented than *S. calcitrans* (2/4). Asia is the second-most taxonomically diverse region for *Stomoxys* spp. [16]. Furthermore, various pathogens have been isolated from various *Stomoxys* spp. in the region [9, 55]. However, only one study was conducted on pathogen transmission by the vector. Three species, *S. transvittatus, S. inornatus*, and *S. omega*, did not report a single positive outcome in transmission of the pathogen by the flies. As this study revealed, various *Stomoxys* spp. transmit certain pathogens at various capacities. For example, *S. n. niger* transmitted the protozoan pathogen *T. evansi* at greater success than *S. calcitrans*. This is also considering that *S. niger* is considered more widely distributed in Africa than *S. calcitrans* [16]. It is also worth noting that while *Stomoxys* spp. are considered general parasites, feeding on a wide variety of hosts, there is documented variation in the genus regarding host association. For example, [16] note that 11 species in this genus are associated with wildlife. Thus, their impact and role in pathogen transmission at the wildlife-livestock-human interface is extremely under-researched.

### Pathogen diversity and distribution

This review showed that *Stomoxys* spp. can transmit a wide variety of microbial pathogens, including protozoa, bacteria, and viruses. The importance of *Stomoxys* spp. as a mechanical vector is well emphasized [1]. A wide range of pathogens have been isolated from *Stomoxys* spp., including those that are vectored by other arthropod vectors. *Babesia* and *Theileria*, which are primarily vectored by ticks, have been isolated from three *Stomoxys* spp., namely, *S. calcitrans, S. indicus*, and *S. pullus* [9, 28, 55]. However, its ability to transmit these diseases has not been well studied. Therefore, *Stomoxys* spp., with their ability to transmit such a wide range of pathogens, may complicate the understanding of the spread, emergence, and re-emergence of diseases vectored by other arthropods. For example, tsetse flies (*Glossina* spp.) are known as the main vectors of *Trypanosoma* pathogens [10, 50]. However, our review findings showed that certain *Stomoxys* spp. can efficiently transmit various *Trypanosoma* species to different hosts. Cherenet *et al.* [10] noted that mechanical transmission can play an important role in the spread of these pathogens, where their main vectors are absent.

There was general variation between regions and continents regarding the pathogens studied, with the only overlap observed between Europe and Asia with regards to LSDV, North America and Africa with regards to *A. marginale*, and Africa and South America with regards to *Trypanosoma* spp. Two protozoan pathogens were demonstrably transmitted by *Stomoxys* spp. *Trypanosoma* spp., causative agents of trypanosomiasis, a variety of diseases affecting humans and animals, including a variety of domestic and wildlife vertebrates, such as surra, nagana, Chagas disease, and African sleeping sickness, are predominantly distributed across Africa, Central and South America, and Asia [14, 50]. *Stomoxys* spp. are considered the most significant vectors of these pathogenic agents after tsetse flies [50]. All the studies on *Trypanosoma* spp. species in this review are from Africa and South America, and various *Stomoxys* spp. demonstrably transmitted the pathogens to different hosts at transmission success rates reaching 100%, highlighting both their prioritisation and threat as risk factors. While *B. besnoiti*, the causative agent that affects a wide variety of domestic and wild animals, has been well documented in Africa and Asia, it has now been established as a re-emerging pathogen in Europe and other parts of the world [40, 52]. Studies of the pathogens in this review were from Europe, and *S. calcitrans* demonstrated the ability to transmit the pathogen in up to 100% of the trials in different hosts. This highlights that this cosmopolitan species poses a risk for re-emerging vector-borne diseases.

Four viral pathogens were demonstrably transmitted by *Stomoxys* spp. The African swine fever virus and the lumpy skin disease virus, two pathogenic agents of notifiable diseases, have recently been introduced into Europe through Eastern Europe and Western Asia [2, 21, 51]. Studies of the pathogens in this review were from Central Asia and Europe, where the diseases are re-emergent and emergent [2, 21, 52] and *S. calcitrans* demonstrably transmitted the pathogens in up to 100% of the attempts, highlighting the flies as a risk factor in emerging and introduced vector-borne diseases. The Porcine Reproductive and Respiratory Syndrome Virus is globally distributed [11]. The study on this pathogen was from the USA, where it reportedly cost the national pig farming industry approximately $560 million annually [52] and was demonstrably transmitted by *S. calcitrans* in up to 100% of the attempts, highlighting the economic risk that *Stomoxys* spp. pose to the livestock industry. The Rift Valley Fever Virus is predominantly distributed across tropical Africa towards the Middle East and is transmitted mainly by mosquito species that occur globally, some of which are found in North America, where its potential introduction has previously been assessed [20, 48]. The study on this pathogen was conducted in North America and was demonstrably transmitted by *S. calcitrans* in over half of the attempted transmissions, thus highlighting the risk this species poses to potentially disseminate emerging zoonoses.

Four bacterial pathogens were demonstrably transmitted by *Stomoxys* spp. *Anaplasma marginale* is distributed along the tropical and subtropical regions, mainly transmitted by ticks, and is one of the most problematic bacterial diseases in agriculture [29]. The two studies in this review were conducted in South Africa and the United States and were demonstrably transmitted to cows by the flies in one of the studies at a moderate transmission success, highlighting the threat posed by *Stomoxys* flies as a passive vector of pathogens. One of the most widespread and persistent pathogens, *S. aureus,* has gained notoriety for its persistent antimicrobial adaptation and, as zoonotic and reverse zoonotic pathogens, poses a huge risk to both human and animal health [54]. In this study, the pathogen was transmitted in North America by *S. calcitrans* in 100% of the attempts, highlighting the potential risk this globally distributed fly species poses at the livestock-human-wildlife interface. *Mycoplasma suis*, the globally distributed bacterial pathogen of pigs and wild porcine [27], was transmitted to pigs by *S. calcitrans* at a low rate. This highlights the potential of the fly species as a health risk factor in the wildlife-livestock interface. *Bacillus anthracis*, the globally distributed zoonotic pathogen [45], was transmitted to guinea pigs at a high rate and to mice at a low rate by *S. calcitrans*. This further highlights the risk this fly species poses to animal health regarding highly infectious diseases. Six studies reported failure of the *Stomoxys* spp. to transmit pathogens at all.

### Host diversity

A total of nine animal hosts were identified in this review. *Stomoxys* spp. have a wide range of host species as general blood-feeding flies, including wild and domestic animals [16, 17, 31, 58, 60]. While *S. calcitrans* is commonly associated with a variety of livestock, it is also associated with wildlife; some species (*S. bilineatus, S. pallidus, S. transvittatus, S. taeniatus, S. inornatus, S. omega,* and *S. varipes*) are mostly associated with wildlife [16]. Furthermore, many of the pathogens isolated from *Stomoxys* have previously been documented in wildlife [3, 9, 12, 20, 31, 36, 40, 46]. *Stomoxys* spp. are known to occasionally bite humans [1, 16]. This study highlighted the wide range of vertebrate hosts that these flies feed on. Most of the studies examined in this review were on livestock and laboratory-reared relatives of wild animals. *Stomoxys* spp. successfully transmitted pathogens to all the host species they were exposed to.

### Detection methods

The distribution of detection methods across the pathogens reported in this review reflects the trends in the literature. Conventionally, protozoan parasites have been detected using microscopy, supported by serological methods [35]. Viral and bacterial pathogens have traditionally been detected using culture methods, inoculation, and serology, as well as microscopy in the case of bacteria [6, 44, 71, 72]. However, these methods have been noted for their low specificity and sensitivity, in addition to being time-consuming, labour-intensive, and requiring high technical expertise [6, 35, 44, 71, 72]. Although these conventional methods are still applied, molecular methods have largely been incorporated in pathogen detection due to alleviating most of the drawbacks of the conventional methods and the universal application [6, 35, 44, 71, 72].

### Pathogen transmission

Transmission experiments reported in this review ranged from biological substrates, host feeding, ingestion by host, injection of vector material, and association of vector to prevalence rates. Field experiments were few and largely yielded inconclusive outcomes. While *Stomoxys* spp. largely yielded at least one positive outcome in transmission of pathogens, the success rates varied widely, from very low (2.4%) to extremely high (100%). The mechanical transmission rate has been shown to vary among *Stomoxys* spp. and with different pathogens, even closely related ones [46]. As mechanical vectors, the flies have demonstrably lower transmission rates than other biological vectors (*e.g.* [62]). In field conditions, this transmission rate can be amplified due to the feeding behaviour of these flies, such as swarming and interrupted feeding [17, 61].

## Conclusion

The lack of diversity in *Stomoxys* spp. research regarding their role in pathogen transmission is concerning, given the relatively high taxonomic diversity of the genus and the wide variety of pathogens they transmit. Future studies should include more species, especially in Africa and Asia, given the high taxonomic diversity in the former and documented evidence of pathogen corridors into Europe in the latter. The livestock-wildlife interface offers an opportunity for biting flies to transmit pathogens between wildlife and livestock [32, 33]. Considering the wide host range of *Stomoxys* spp., including wildlife, future research should pay more attention to wildlife species. More focus is also needed on the dispersal capabilities of the flies, especially given the variation in transmission after exposure to pathogens. This review primarily focused on studies linking the pathogen to the host and vector, missing the opportunity to assess a broader range of pathogens carried by *Stomoxys* spp. and their prevalence in the flies. Most studies centred on *S. calcitrans*, resulting in limited data on the pathogens transmitted by other species. The variability in methods, although expected given the type of research, hindered meta-analyses and prevented a more comprehensive understanding of pathogen transmission by these flies. The exclusion of non-English articles was also a missed chance to evaluate a wider geographic scope. The studies showed a broad sampling range with rather large standard deviations concerning both vectors and hosts.

## Supplementary material

*Supplementary Table S1*: List and distribution of *Stomoxys* species worldwide (Duvallet & Hogsette, 2023) *Supplementary Table S2*: The Joanna Briggs Institute (JBI) critical appraisal checklist for studies reporting on experimental transmission of microbial pathogens by *Stomoxys* spp. *Supplementary Table S3*: Characteristics of the eligible studies on experimental transmission of microbial pathogens by *Stomoxys* spp. *Supplementary Table S4*: Summary statistics of studies on the transmission of microbial pathogens by *Stomoxys* spp. The supplementary material of this article is available at https://www.parasite-journal.org/10.1051/parasite/2026014/olm.
