# Supplementary material for: A systematic review of experimental evidence on microbial pathogen transmission by Stomoxys spp
Source: Parasite. 2026 Mar 19;33:13. doi: 10.1051/parasite/2026014 (PMC13001615; doi:10.1051/parasite/2026014)
Supplement: Supplementary file 1 — Supplementary Table S1: List and distribution of Stomoxys species worldwide (Duvallet & Hogsette, 2023) [file parasite-33-13-s1.pdf]

Supplementary Table S1. List and distribution of *Stomoxys* species worldwide (Duvallet & Hogsette, 2023)

Species	Nearctic	Palearctic	Indomalaya	Neotropical	Afrotropical	Oceania	Reunion
Stomoxys calcitrans	Yes	Yes	Yes	Yes	Yes	Yes	Yes
Stomoxys sitiens	No	No	Yes	No	Yes	No	No
Stomoxys omega	No	No	No	No	Yes	No	No
Stomoxys niger	No	No	No	No	Yes	No	Yes
Stomoxys xanthomelas	No	No	No	No	Yes	No	No
Stomoxys pallidus	No	No	No	No	Yes	No	No
Stomoxys ochrosoma	No	No	No	No	Yes	No	No
Stomoxys luteolus	No	No	No	No	Yes	No	No
Stomoxys stigma	No	No	No	No	Yes	No	No
Stomoxys transvittatus	No	No	No	No	Yes	No	No
Stomoxys boueti	No	No	No	No	Yes	No	No
Stomoxys taeniatus	No	No	No	No	Yes	No	No
Stomoxys inornatus	No	No	No	No	Yes	No	No
Stomoxys varipes	No	No	No	No	Yes	No	No
Stomoxys indicus	No	No	Yes	No	No	No	Yes
Stomoxys bengalensis	No	No	Yes	No	No	No	No
Stomoxys pullus	No	No	Yes	No	No	No	No
Stomoxys uruma	No	No	Yes	No	No	No	No
