# Supplementary material for: A systematic review of experimental evidence on microbial pathogen transmission by Stomoxys spp
Source: Parasite. 2026 Mar 19;33:13. doi: 10.1051/parasite/2026014 (PMC13001615; doi:10.1051/parasite/2026014)
Supplement: Supplementary file 2 — Supplementary Table S2: The Joanna Briggs Institute (JBI) critical appraisal checklist for studies reporting on experimental transmission of microbial pathogens by Stomoxys spp. [file parasite-33-13-s2.pdf]

A systematic review of experimental evidence on pathogen transmission by Stomoxys spp: pathogen diversity and host range

Supplementary Table S2: The Joanna Briggs Institute (JBI) critical appraisal checklist for studies reporting on experimental transmission of microbial pathogens by *Stomoxys* spp.

	References	1	2	3	4	5	6	7	8	9	Total	Score
**1**	Balmos *et al*., 2023	Y	Y	Y	Y	Y	N	N	Y	N	6/9	66.6%
**2**	Burg *et al.,* 1994	Y	Y	Y	Y	Y	Y	Y	Y	Y	9/9	100.0%
**3**	Buxton *et al.,* 1985	Y	Y	Y	Y	Y	N	N	Y	Y	6/9	66.6%
**4**	Castro *et al*., 2016	Y	Y	Y	Y	Y	N	N	Y	N	6/9	66.6%
**5**	Foil *et al*., 1983	Y	Y	N	Y	Y	N	Y	Y	Y	7/9	77.8%
**6**	Gibbs *et al.,* 1973	Y	Y	N	Y	Y	N	Y	Y	Y	7/9	77.8%
**7**	Haegeman *et al.,* 2023	Y	Y	Y	Y	Y	Y	Y	Y	Y	9/9	100.0%
**8**	Hawkins *et al.,* 1973	Y	Y	N	Y	Y	Y	Y	Y	Y	9/9	100.0%
**9**	Heller *et al.,* 2024	Y	Y	Y	Y	Y	Y	Y	Y	Y	9/9	100.0%
**10**	Hemida *et al*., 2022	Y	Y	Y	Y	Y	Y	Y	Y	Y	9/9	100%
**11**	Issimov *et al*., 2020	Y	Y	N	Y	Y	Y	Y	Y	Y	8/9	88.9%
**12**	Kitching & Mellor, 1987	Y	Y	N	N	Y	N	Y	Y	Y	6/9	66.6%
**13**	Mellor *et al*., 1987	Y	Y	N	N	Y	Y	N	Y	Y	6/9	66.6%
**14**	Lendzele *et al*., 2019	Y	Y	Y	Y	Y	Y	Y	Y	N	8/9	88.9%
**15**	Liénard *et al*., 2017	Y	Y	N	N	Y	N	Y	Y	Y	6/9	66.6%
**16**	Mihok *et al*., 1995	Y	Y	Y	Y	Y	Y	Y	Y	Y	9/9	100.0%
**17**	Nayani *et al*., 2024	Y	Y	Y	Y	Y	Y	Y	Y	Y	9/9	100%
**18**	Ngerwana & Kilelo, 1994	Y	Y	N	N	Y	Y	Y	Y	Y	7/9	77.8%
**19**	Olesen *et al*., 2018	Y	Y	N	Y	Y	Y	Y	Y	Y	8/9	88.9%
**20**	Potgierter *et al*., 1981	Y	Y	N	Y	Y	Y	Y	Y		8/9	88.9%
**21**	Prullage *et al.,* 1993	Y	Y	N	Y	Y	Y	Y	Y	Y	8/9	88.9%
**22**	Rochon *et al.,* 2011	Y	Y	Y	Y	Y	Y	Y	Y	Y	9/9	100%
**23**	Scoles *et al.,* 2005	Y	Y	Y	Y	Y	Y	Y	Y	Y	9/9	100%

**24**	Sharif *et al.*, 2017	Y	Y	Y	N	Y	N	Y	Y	Y	8/9	88.9%
**25**	Sharif *et al.,* 2019	Y	Y	N	N	Y	N	Y	Y	Y	7/9	77.8%
**26**	Sohier *et al.*, 2019	Y	Y	Y	Y	Y	Y	Y	Y	Y	9/9	100%
**27**	Sumba *et al.*, 1998	Y	Y	Y	Y	Y	Y	Y	Y	Y	9/9	100%
**28**	Turell & Knudson, 1987	Y	Y	N	Y	Y	Y	Y	Y	Y	8/9	88.9%
**29**	Turell *et al.*, 2010	Y	Y	N	N	Y	Y	Y	Y	Y	7/9	77.8%
**30**	Weber *et al.*, 1988	Y	Y	N	Y	Y	Y	Y	Y	Y	8/9	88.9%

<u>The checklist questions to determine the risk of bias for the included studies</u>:

(a) Was the title and abstract of the study relevant?

(b) Were the methods and data analysis of the study clear and sufficiently covered?

(c) Does the study give a detailed description of the statistical approach and analysis?

(d) Was the sample size acceptable?

(e) Was the interpretation of the results and discussion taking into account the aim/objectives of the study?

(f) Were the vector and host samples randomly selected from the population?

(g) Were the vector, host and pathogen/s clearly identified and allocated

(h) Was the study/ experimental design ethically acceptable?

(i) Were the transmission outcomes clearly stated?
