# Supplementary material for: A systematic review of experimental evidence on microbial pathogen transmission by Stomoxys spp
Source: Parasite. 2026 Mar 19;33:13. doi: 10.1051/parasite/2026014 (PMC13001615; doi:10.1051/parasite/2026014)
Supplement: Supplementary file 3 — Supplementary Table S3: Characteristics of the eligible studies on experimental transmission of microbial pathogens by Stomoxys spp. [file parasite-33-13-s3.pdf]

Supplementary Table S3. Characteristics of the eligible studies on experimental transmission of microbial pathogens by *Stomoxys* spp.

**Vector Species**	**Vector Sample size**	**Experimental Design**	**Host**	**Host Sample size**	**Pathogen type**	**Pathogen**	**Detection method**	**Country**	**Region**		**Outcome (Yes/ No/ NA)**	**Transmission success (time after next meal)**	**Other vectors present**	**Reference**
S. calcitrans	99	Association	Pig	N/A	Virus	African Swine Fever Virus	Molecular (Real-time PCR)	Romania	Eastern Europe	Europe	NA	NA	Yes	[2]
S. calcitrans	60	Injection by fly material, Feeding on host	Mouse	6	Bacteria	*Ehrlichia risticii*	Microscopy (IFA)	USA	North America	North America	No	0% (0h)	No	[4]
S. calcitrans	639	Injection by fly material, Feeding on host	Sheep	10	Virus	Bovine leukosis virus	Serology (AGID)	N/A	N/A	N/A	Yes	70% (30 min, injection), 0% (1h - 2.5h), 0% (feeding)	Yes	[5]
			Cow	8	Virus	Bovine leukosis virus					Yes			
S. calcitrans	200	Association	Cow	N/A	Bacteria	*Escherichia coli*	Molecular (RAPD)	Brazil	South America	South America	No	N/A	No	[7]
S. calcitrans	253	Feeding on host	Horse	3	Virus	Equine infectious anemia virus	Serology (AGID)	N/A	N/A	N/A	Yes	66.66% (N/A)	Yes	[18]
S. calcitrans	10	Feeding on cultured medium	Cell culture (Cow)	10	Virus	Bovine herpes mammilities	Culture (Titration)	N/A	N/A		Yes	90% (0.5 h)	No	[19]
S. calcitrans	3630	Feeding on host	Cow	26	Virus	Lumpy skin disease virus	Serology (IPMA) , ELISA), Culture (VNT), Molecular (qPCR)	Belgium	Western Europe	Europe	Yes	15.4% (N/A)	No	[21]

S. calcitrans	1 097	Feeding on host	Horse	10	Virus	Equine infectious anemia virus	Serology (Immunodiffusion test)	United States	North America	North America	No	0% (1h, 3d)	Yes	[23]
S. calcitrans	33 000	Feeding on host	Cow	18	Protozoa	*Trypanosoma vivax*	Molecular (PCR), Microscopy (Woo, Brener), Hematology (PCV), Serology (immonoteste, ELISA, IFAT)	Brazil	South America	South America	Yes	100% (NA)	No	[24]
S. calcitrans	81	Association	Camel	52	Virus	Middle East respiratory syndrome coronavirus	Molecular (qPCR)	N/A	Middle East	Asia	No	0%	Yes	[25]
S. calcitrans, S. sitiens, S. indica	1280	Feeding on host	Cow	6	Virus	Lumpy skin disease virus	Serology (Serum neutralization test), Culture (Titration), Molecular (PCR)	Kazakhstan	Central Asia	Asia	Yes	83.33% (N/A)	No	[30]
S. calcitrans	200	Feeding on host	Sheep	2	Virus	Capripox virus	Culture (Titration)	England	Western Europe	Europe	Yes	50%	Yes	[34]
S. calcitrans	107	Feeding on host	Goat	2	Virus	Capripox virus	Culture (Titration)	N/A	N/A	N/A	Yes	50% (24h),	No	[42]
			Pig	2	Virus	African Swine fever virus					Yes	100% (1h, 24h), 0% (2 days, 4, days, 6 days)		
S. niger niger, S. omega	297	Association	Cow	N/A	Virus	Foot-and-mouth disease virus	Molecular (RT-qPCR)	Cameroon	West Africa	Africa	10	NA	Yes	[37]

S. calcitrans	1 000	Feeding on blood substrate, Feeding on host	Sterile blood	3	Protozoa	*Besnoitia besnoiti*	Molecular (qPCR), Serology (modified fluorescent antibody test)	France	Western Europe	Europe	Yes	100% (0-24h)	No	[38]
			Cow	1	Protozoa	*Besnoitia besnoiti*					Yes			
Stomoxys niger bilineatus, S. n. niger, S. pallidus, S. transvittatus, S. taeniatus, S. inornatus, S. calcitrans and S. varipes	441	Feeding on host	Mouse	177	Protozoa	*Trypanosoma brucei, Trypanosoma congolense, Trypanosoma evansi, Trypanosoma vivax,*	Microscopy (wet smear)	Kenya	East Africa	Africa	Yes	Stomoxys *niger bilineatus* (5.5%, 0%, 0%, 2.4%), *S. n. niger* (0%, 0%, 0%, 14.3), *S. pallidus* (100%, 0%, 0%, 0%), *S. transvittatus* (0%), *S. taeniatus* (26.7%, 0%, 0%, 0%), *S. inornatus* (0%), *S. calcitrans* (0%) and *S. varipes* (16.1%, 0%, 100%, 0%)	Yes	[43]
S. calcitrans	48	Feeding on blood substrate	Sterile blood	48	Bacteria	*Staphylococcus aureus*	Culture, Molecular (PCR)	Canada	North America	North America	Yes	100% (0-24h)	No	[47]

S. calcitrans	N/A	Feeding on host	Goat	2	Protozoa	*Trypanosoma evansi*	Microscopy (Wet smear), Hematology (HCT)	Kenya	East Africa	Africa	No	0% (NA, 48h)	No	[48]
			Camel	2	Protozoa	*Trypanosoma evansi*					No			
S. calcitrans	160	Ingestion by host	Pig	8	Virus	African Swine Fever Virus	Culture (Titration), Serology (Elisa), Molecular (qPCR)	Denmark	Western Europe	Europe	Yes	87.5%	No	[51]
S. calcitrans	~440	Feeding on host	Cow	6	Bacteria	*Anaplasma marginale*	N/A	South Africa	Southern Africa	Africa	Yes	33.333% (N/A)	Yes	[56]
S. calcitrans	242	Feeding on host	Pig	15	Bacteria	*Eperythrozoon suis*	N/A	N/A	N/A	N/A	Yes	20% (0h), 0% (1h)	Yes	[57]
S. calcitrans	180	Feeding on host	Pig	20	Virus	Porcine Reproductive and Respiratory Syndrome Virus	Culture (Titration), Molecular (qPCR)	USA	North America	North America	Yes	100%(0-24h)	No	[59]
S. calcitrans	66	Feeding on host	Cow	4	Bacteria	*Anaplasma marginale*	Microscopy (Blood smears), Serology (ELISA), Molecular (PCR)	USA	North America	North America	No	0% (0h)	Yes	[62]
S. calcitrans	2400	Feeding on blood substrate	Cow	2	Protozoa	*Besnoitia besnoiti*	Microscopy, Serology, Molecular (Real-time PCR)	France	Western Europe	Europe	Yes	0% (48h) to 100% (0h)	No	[63]
S. calcitrans	300	Feeding on host	Rabbit	3	Protozoa	*Besnoitia besnoiti*	Molecular (Western blot), Serology (Immunofluorescence Assay)	France	Western Europe	Europe	Yes	30%(NA)	No	[64]
S. calcitrans	4880	Feeding on host	Cow	14	Virus	Lumpy skin disease virus	Culture (Viral neutralization test), Molecular (qPCR)	Belgium	Westen Europe	Europe	Yes	35.7%	Yes	[66]

S. niger niger, S. taeniatus	355	Feeding on host	Mouse	555	Protozoa	*Trypanosoma evansi, Trypanosoma congolense*	Microscopy (wet smears)	Kenya	East Africa	Africa	Yes	15.4% *(S. taeniatus, T. evans*i), 0% (S.taeniatus, *T. congolense*), 23.9% (*S. niger niger*, *T. evans*i), 6.9% (S*. niger niger, T. congolense*),	No	[67]
S. calcitrans	28	Feeding on host	Guinea pig	8	Bacteria	*Bacillus anthracis*	Culture (Biochemical test), Microscopy	N/A	N/A	N/A	Yes	75% (1h), 100% (4h), 0% (24h)	Yes	[69]
			Mouse	8	Bacteria	*Bacillus anthracis*					Yes	12.5% (1h)		
S. calcitrans	7	Feeding on host	Mouse	NA	Virus	Rift Valley Fever Virus	Culture (Titration)	USA	North America	North America	Yes	57% (20 min)	Yes	[70]
S. calcitrans	NA	Feeding on host	Cow	27	Virus	Bovine leukemia virus	Serology (AGID), Microscopy (lymphocyte examination)	N/A	N/A	N/A	NA	N/A	No	[73]
