# Supplementary material for: A systematic review of experimental evidence on microbial pathogen transmission by Stomoxys spp
Source: Parasite. 2026 Mar 19;33:13. doi: 10.1051/parasite/2026014 (PMC13001615; doi:10.1051/parasite/2026014)
Supplement: Supplementary file 4 — Supplementary Table S4: Summary statistics of studies on the transmission of microbial pathogens by Stomoxys spp. [file parasite-33-13-s4.pdf]

Supplementary Table 4. Summary statistics of studies on the transmission of microbial pathogens by *Stomoxys* spp.

	N	Percentage
**Study Design (In vivo/ In vitro)**		
**In vitro**	2	7
**In vitro/ In vivo**	1	3
**In vivo**	27	90
**Experimental Design**		
**Association**	4	13
**Feeding on blood substrate**	3	10
**Feeding on cultured medium**	1	3
**Feeding on host**	22	73
**Ingestion by host**	1	3
**Injection by fly material,**	2	7
**Host**		
**Camel**	2	7
**Cell culture (Cow)**	1	3
**Cow**	11	31
**Goat**	2	7
**Guinea pig**	1	3
**Horse**	2	7
**Mouse**	5	17
**Pig**	5	17
**Rabbit**	1	3
**Sheep**	2	7
**Sterile blood**	2	7
**Outcome (Yes/ No/ NA)**		
**NA**	3	10

**No**	6	20				
**Yes**	21	70				
**Pathogen type (Virus/ Bacteria/ Protozoal)**						
**Bacteria**	7	23				
**Protozoa**	7	23				
**Virus**	16	54				
**Detection method**			Virus	Protozoa	Bacteria	Total
**Molecular**	15	50	8	4	3	15
**Microscopy**	9	30	1	5	3	9
**Serology**	12	40	8	4	1	13
**Haematology**	2	7	0	2	0	2
**Culture**	11	37	9	0	2	11
**Total**			26	15	9	50
